# Impact of Early Rejection Treatment on Infection Development in Kidney Transplant Recipients: A Propensity Analysis

**DOI:** 10.1155/2024/6663086

**Published:** 2024-03-01

**Authors:** Simran Gupta, Juan Gea-Banacloche, Raymond L. Heilman, Reena N. Yaman, Hay Me Me, Nan Zhang, Holenarasipur R. Vikram, Lavanya Kodali

**Affiliations:** ^1^Division of Infectious Diseases, Department of Medicine, Massachusetts General Hospital, Boston, MA, USA; ^2^Harvard Medical School, Boston, MA, USA; ^3^Division of Clinical Research, National Institute of Allergy and Infectious Diseases, Bethesda, Maryland, USA; ^4^Division of Nephrology, Mayo Clinic Arizona, Phoenix, Arizona, USA; ^5^Transplant Center, Mayo Clinic Arizona, Phoenix, Arizona, USA; ^6^Department of Internal Medicine, Mayo Clinic Arizona, Phoenix, Arizona, USA; ^7^Department of Quantitative Health Sciences, Mayo Clinic Arizona, Phoenix, Arizona, USA; ^8^Division of Infectious Diseases, Mayo Clinic Arizona, Phoenix, Arizona, USA

## Abstract

**Introduction:**

The impact of renal allograft rejection treatment on infection development has not been formally defined in the literature.

**Methods:**

We conducted a retrospective cohort study of 185 rejection (case) and 185 nonrejection (control) kidney transplant patients treated at our institution from 2014 to 2020 to understand the impact of rejection on infection development. Propensity scoring was used to match cohorts. We collected data for infections within 6 months of rejection for the cases and 18 months posttransplant for controls.

**Results:**

In 370 patients, we identified 466 infections, 297 in the controls, and 169 in the cases. Urinary tract infections (38.9%) and cytomegalovirus viremia (13.7%) were most common. Cumulative incidence of infection between the case and controls was 2.17 (CI 1.54–3.05); *p* < 0.001. There was no difference in overall survival (HR 0.90, CI 0.49–1.66) or graft survival (HR 1.27, CI 0.74–2.20) between the groups. There was a significant difference in overall survival (HR 2.28, CI 1.14–4.55; *p* = 0.019) and graft survival (HR 1.98, CI 1.10–3.56; *p* = 0.023) when patients with infection were compared to those without.

**Conclusions:**

As previously understood, rejection treatment is a risk factor for subsequent infection development. Our data have defined this relationship more clearly. This study is unique, however, in that we found that infections, but not rejection, negatively impacted both overall patient survival and allograft survival, likely due to our institution's robust post-rejection protocols. Clinicians should monitor patients closely for infections in the post-rejection period and have a low threshold to treat these infections while also restarting appropriate prophylaxis.

## 1. Introduction

Renal transplant rejection is an immunological response in a recipient to recognition of foreign antigen in an allograft [1]. Acute allograft rejection can occur as early as one week posttransplant, with the highest risk in the first three months [2]. Acute T-cell mediated rejection (TCMR) is due to cytotoxic T-cell reaction to human leukocyte antigens (HLA) on the donor kidney [3]. Acute antibody-mediated rejection (AMR) is a result of the interaction between donor specific antibodies (DSA) and mismatched HLA or non-HLA molecules on the surface of allograft endothelial cells and leads to allograft injury usually by way of complement pathway activation [4]. Banff scoring is used to grade rejection based on histologic findings. Acute TCMR is graded into borderline, IA, IB, IIA, IIB, and III depending on the severity of inflammation in the tubules and arteries. Acute AMR is diagnosed if biopsy shows features of acute endothelial damage presenting as glomerulitis (g), peritubular capillaritis (ptc) [*g* + ptc ≥ 2] and/or thrombotic microangiopathy, unexplained acute tubular necrosis, evidence of recent antibody interaction with vascular endothelium such as the presence of C4d staining which is a marker of complement activity, and/or serologic evidence of circulating DSAs [5].

Most recent data from the Organ Procurement and Transplantation Network (OPTN) suggest that the incidence of acute renal allograft rejection in adult kidney transplant patients within the first year of transplant is as high as 7.0%, with the highest percentage of patients experiencing rejection aged 18 to 34 years [6]. Risk factors for allograft rejection include older age of donor, younger age of recipient, pretransplant pregnancies or exposure to blood products (higher panel reactive antibody levels), immunologic incompatibility, longer cold ischemia time, and delayed graft function [7, 8]. OPTN also noted higher rates of acute rejection in patients who had received interleukin-2 receptor antibody induction compared to T-cell-depleting induction.

Acute allograft rejection has previously been noted by multiple studies to be associated with subsequent graft loss [7, 9, 10], with factors including timing of rejection, number of acute rejection episodes, and degree of kidney function recovery after rejection treatment all affecting long-term outcomes [11]. Infections in kidney transplant recipients are also known causes of morbidity and mortality, with prior research showing that infection is the second leading cause of death in recipients with allograft function [12–14]. However, the relationship between rejection and its treatment on subsequent infection development in transplant recipients is not defined, though has been hypothesized. In this proof-of-concept study, we aim to compare kidney transplant recipients who were and were not treated for allograft rejection and understand the impact on infection development, overall survival, and graft survival.

## 2. Methods

This study was approved by the Mayo Clinic Institutional Review Board, and written informed consent was waived. We conducted a retrospective chart review of all adult kidney transplant (KT) recipients who have undergone treatment for rejection at our institution from January 1, 2014, to December 31, 2020. Inclusion criteria included any KT recipient with at least one episode of rejection (and subsequent treatment) within 1 year, or 12 months, of transplantation. We included any type of acute rejection receiving treatment, including acute TCMR, AMR, mixed, and borderline rejections. Patients with rejection episodes more than 1 year after transplant were excluded. Data from all episodes of rejection within the first year after transplant for each patient were gathered, but only the first episode of rejection was used in the analysis. Using a previously amassed database of all patients who had undergone kidney transplant at our institution during the same time period, we used propensity-score matching to match the above cohort (case) to a similar cohort of patients who were not diagnosed with allograft rejection (control).

Baseline data abstracted from the Electronic Health Record (EHR) included demographics and comorbidities, pretransplant coccidioidomycosis status, transplant data including source of graft, PHS-increased risk, hepatitis B and C status of the donor and recipient, Epstein–Barr virus (EBV) and Cytomegalovirus (CMV) serology status, and immunosuppression and prophylaxis regimens. We also identified the estimated glomerular filtration rate (eGFR), defined as the laboratory values at approximately 4 months posttransplant for the case group and control group. We collected data for each episode of rejection within 12 months of transplant including the type of rejection therapy, immunosuppressant trough level at rejection, and laboratory values at rejection.

We also gathered data for each patient infection within 6 months of rejection treatment for the case cohort and up to 18 months after transplant for the control cohort (assuming a maximum 12 month period for rejection in the case cohort + up to 6-month period for subsequent infection), yielding a potential duration of follow-up of 18 months for both cases and controls. This included the type of infection and labs at the time of identification of infection. Infections were identified by reviewing clinical notes in the EHR as well as by reviewing each patient's microbiological data. Infections prior to rejection in the case cohort were not counted towards our primary outcome because we were investigating how rejection impacts infection. Additionally, infections that initially occurred prior to rejection treatment but recurred after infection treatment were excluded. Also, based on a thorough chart review, patients who had recurrent UTI's prior to transplant due to conditions such as neurogenic bladder or reflux nephropathy were excluded prior to matching so as to not confound infection risk.

Infections overall were defined as positive microbiological testing (i.e., blood, body fluid, and tissue culture) along with clinical infectious symptoms. Episodes of asymptomatic bacteriuria were excluded. We also aimed to specifically identify opportunistic infections (OIs). OIs were defined as those that cause disease when the immune system is impaired but are not typically pathogenic in healthy persons [15]. We used a previous definition for OI defined by Fishman that includes “pneumocystis pneumonia; herpesvirus infections including herpes simplex virus, varicella-zoster, CMV, and EBV; infections with Listeria, Nocardia, Toxoplasma, Strongyloides, Leishmania, Trypanosoma cruzi; polyomavirus BK nephropathy and viremia; JC polyomavirus infection; Cryptococcus neoformans infection; Mycobacterium tuberculosis or atypical mycobacteria infection; and infection with aspergillus, atypical molds, mucor species.” [16]. Invasive coccidioidomycosis was also included in the OI category given that our institution is in an endemic location for this fungal infection. Sars-CoV-2 infection was included in the category of “pneumonia requiring admission.”

The primary outcome was the cumulative incidence of infection in each cohort. Other outcome data collected included death at any point and graft loss within 6 months of infection. Outcome labs were collected at 1-year posttransplant. We compiled and managed our data using the REDCap (Research Electronic Data Capture) tool supported at our institution.

### 2.1. Statistical Analysis

The propensity score was used for cohort matching. The propensity score is the probability of patients being in the rejection group conditional on observed characteristics (age, sex, diabetes, BMI, transplant year, donor type, induction therapy, steroid avoidance, and eGFR at 4 months), chosen based on data that these variables are independent risk factors for rejection [7, 17–21]. The propensity score allows us to design and analyze a nonrandomized study so that the observed characteristics will be similar between the rejection and nonrejection patients. Studies have shown that propensity score matching eliminates a greater proportion of the systematic differences in clinical characterizes between rejection and nonrejection subjects compared to other propensity score methods (i.e., stratification on the propensity score or covariate adjustment using the propensity score) [22]. Of note, we specifically chose to match eGFR at 4 months to make sure that we captured the impact of infection on graft function accurately by starting off at a comparable eGFR between the two cohorts. The standardized difference is usually used to compare the mean of continuous variables and prevalence of categorical variables to assess the balance of covariates between the two groups before and after propensity score match. From Supplemental Figure 1, we can see that the propensity score matching reduces the imbalance between the two groups in our study to a large degree, especially for the year of transplant, eGFR at 4 months, and steroid avoidance. After matching, two sample *t*-test or chi-square test was used to compare the demographics and clinical characteristics between the cases and controls.

To investigate how rejection impacted overall survival, graft survival, and infection status, Cox regression with time-dependent covariate was used, in which rejection status was treated as the time-dependent covariate to avoid immortal bias [23]. In this model, before a patient developed infection, they were considered in the risk set of the nonrejection cohort, but after they developed rejection, they were switched to the risk set of the rejection cohort as their exposure to rejection had started. The use of the Cox regression model allowed us to incorporate the time-to-event information to estimate cumulative incidence accurately. For the rejection patients who had infections prior to their rejection, the data from the infections prior to rejection were not included as outcomes, but the patients were not excluded. In patients who had more than one episode of infection, only the first episode of infection was used in the propensity analysis, though data from all infections within the appropriate time period were gathered and are reported in the results. Similarly, to investigate how infection impacted overall survival and graft survival, Cox regression with time-dependent covariate was used, in which infection status was treated as the time-dependent covariate. All analysis was performed using R version 4.1.2 (R Foundation for Statistical Computing, Vienna, Austria). 0.05 was chosen as the cut-off criterion for statistical significance.

### 2.2. Immunosuppression, Rejection Treatment, and Prophylaxis Protocols

Our standard infection prophylaxis after KT includes trimethoprim-sulfamethoxazole (TMP/SMX) for 6 months and fluconazole for one year if negative coccidioides serology and no evidence of previous active coccidioidal infection. Patients with positive coccidioides serology and prior active coccidioides infection receive lifelong prophylaxis with fluconazole. For high-risk CMV recipients with mismatch serology (CMV D+/R−), valganciclovir is given for 6 months. We maintain valganciclovir for 3 months for patients with intermediate risk serology (CMV D+/R+ or CMV D−/R+) or acyclovir for 1 month in low-risk (CMV D−/R−) patients. Treatment for acute AMR includes intravenous (IV) methylprednisolone 3–5 milligram/kilogram for 3 days and plasmapheresis (7–10 sessions) with intravenous immunoglobulin (IVIG) 0.1 gram/kilogram after each plasmapheresis (PLEX) session, with a large dose of 2 gram/kilogram at the end of PLEX. Eculizumab is considered case-by-case, based on severity of AMR and presence of thrombotic microangiopathy. Rituximab and bortezomib are occasionally used for treatment of AMR. Treatment for ATCMR includes IV methylprednisolone or oral steroids and ATG for Banff IIA and above. For both AMR and ATCMR, immunosuppression is optimized and steroids are added to the maintenance regimen if patients were not already on it.

When lymphocyte-depleting agents or IL-2 inhibitors (basiliximab) are used for induction therapy, viral prophylaxis for CMV is based on the recipient's seropositivity. For CMV mismatch (D+/R−), 6 months of prophylaxis with valganciclovir is used. For intermediate risk (R+), 3 months of prophylaxis with valganciclovir is used. For low-risk patients (D−/R−), one month of acyclovir is used as prophylaxis against HSV. We used trimethoprim/sulfamethoxazole for six months as prophylaxis against PJP pneumonia. Fluconazole 100 mg is used as prophylaxis for 12 months in all patients with negative coccidioides serologies. Patients with a previous history of coccidioidomycosis in the 12 months prior to transplant or with positive coccidioides serology receive 400 mg of fluconazole for the first year posttransplant, followed by 200 mg for life.

Following rejection therapy, for lymphocyte-depleting agents or pulse corticosteroid treatment of rejection, TMP/SMX SS is reinitiated for 3 months. Coccidioides serology is repeated and if still seronegative, 3 months of prophylaxis with fluconazole is restarted. For pulse corticosteroid therapy for rejection, high-risk CMV recipients are started on prophylaxis with valganciclovir for 4 weeks. For lymphocyte-depleting therapy, valganciclovir is reinitiated for 6 months in high-risk CMV status, 3 months in intermediate risk, and acyclovir is used for one month in low-risk patients.

## 3. Results

From January 1, 2014, to December 31, 2020, we identified 185 kidney transplant patients who were diagnosed with and underwent treatment for acute allograft rejection and 185 kidney transplant patients who did not. The mean age at transplant was 53.6 and 53.0 years old for the case and control cohorts, respectively. Male patients made up 55.7% and 57.8% of the case and control cohorts, respectively. eGFR at four months posttransplant was similar for both cohorts (49.7 vs. 49.5, *p* = 0.935).  Table 1 depicts the demographics and comorbidities for these cohorts of patients, after propensity score matching (Table 1). There were no significant differences in baseline demographics and comorbidities between the two cohorts of patients.

Of the 185 patients in the rejection (case) cohort, 149 (80.5%) had acute TCMR, 27 (14.6%) had AMR, and 9 (4.9%) had mixed rejection. Of the patients with acute TCMR, 64 (43.0%) had borderline rejection, 34 (22.8%) were Banff 1a, 15 (10.1%) were Banff 1b, 27 (18.1%) were Banff IIa, and 9 (6.0%) were Banff IIb. IV steroids were the most common rejection treatment (*n* = 173, 93.5%). 34 (18.4%) patients received IVIG, 27 (14.6%) received PLEX, and 6 (3.2%) received rituximab for rejection treatment. Baseline immunosuppression regimen was intensified in 11 (5.9%) patients. Median time to first rejection was 340 (95% CI: 249, 457) days. There were a few patients who had multiple rejections within the first year posttransplant. 162 (87.5%) patients had one episode of rejection, 19 (10.3%) had two episodes, and 4 (2.2%) had three episodes of rejections.

A total of 466 infections were identified in this study. Median time to first infection was 41 days posttransplant in the case cohort and 457 days posttransplant in the control cohort. In the control cohort, we found 297 total infections in 130 patients over 18 months of posttransplant.

**Figure 1:**
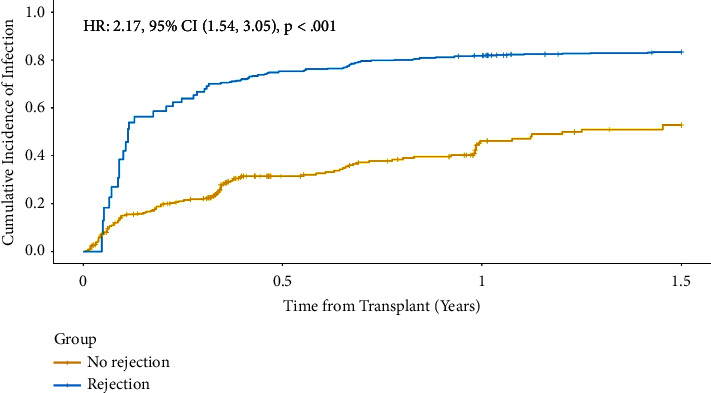
Cumulative incidence of infection by rejection status. There was a statistically significant difference in the cumulative incidence of infection between the rejection and nonrejection cohorts. Rejection patients were 2.17 times more likely to develop infections than nonrejection patients (*p* < 0.001).

Among the rejection cohort, we identified 169 total infections in 79 total patients within six months of rejection treatment. Of these, we noted 46 OIs in the case cohort and 95 in the control cohort.  Table 2 depicts the breakdown of all infections identified. A few patients had more than one sequential infection within their respective follow-up periods. For the 79 rejection patients with infections, 69 (87.3%) only had one type of infection, 9 (11.4%) had two types of infections, and 1 patient (1.3%) had three different infections. For the 130 nonrejection patients with infections, 115 (88.5%) had one type of infection, 13 (10.0%) had two types of infections, and 1 patient (0.8%) had three different infections. The calculated cumulative incidence of infection, using time-varying Cox regression and the hazard ratios between the rejection and nonrejection groups, was 2.17 (95% CI 1.54–3.05); *p* < 0.001. The infection incidence rate for the cases was 9.63 (95% CI: 7.62 to 12.0) per 100 person-months and for the controls was 5.5 (95% CI: 4.59 to 6.53) per 100 person-months. The incidence rate ratio was 1.75 (95% CI: 1.31 to 2.34), which shows that the rejection patients had a significantly higher incidence rate of infection compared to the nonrejection patients.  Figure 1 presents the cumulative incidence between the two cohorts of patients.

We also performed a multivariate Cox regression model (again treating rejection as a time-dependent covariate) to analyze time to infection in order to more rigorously control for potential confounding variables and accurately discern the independent effect of rejection on infection. The model showed that after adjusting for the other covariates, rejection was still significantly associated with a higher risk of developing infection (HR: 2.11, 95% CI: 1.49 to 2.99, *p* < 0.001).

Most common infections identified were symptomatic urinary tract infections (28.9% and 32.6% of case and control patient infections, respectively), CMV viremia (23.3% and 16.0% of case and control patient infections, respectively), and BK viremia (5.6% and 18.8% of case and control patient infections, respectively). Among the 370 patients, 93 had CMV mismatch, and 23 (24.7%) developed CMV viremia; 277 did not have CMV mismatch and 41 (14.8%) of those patients developed CMV viremia. CMV mismatch was a risk factor for CMV viremia (*p*=0.028). Additionally, there was no difference in CMV incidence between induction agents. There were no significant differences in fungal infections between the cohorts.

**Figure 2:**
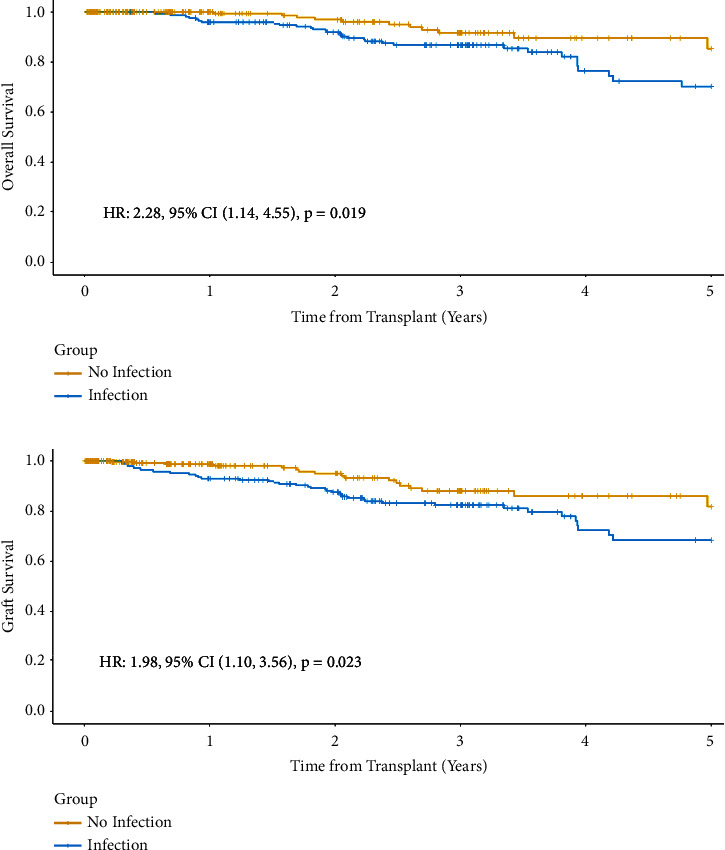
Overall patient survival and graft survival by infection status. (a) Patients without infections had better overall survival than patients with infections (*p*=0.019). (b) Patients without infections had higher rates of allograft survival than patients with infections (*p*=0.023).

We used Cox regression with a time-dependent covariate model to evaluate the impact of rejection treatment on overall survival and graft survival. We found that rejection did not impact overall survival (HR 0.90, 95% CI 0.49–1.66, *p*=0.70) or graft survival (HR 1.27, 0.74–2.20, *p*=0.40). However, when we used Cox regression with a time-dependent covariate model to evaluate the impact of infections on overall patient survival and graft survival in the kidney transplant population, we found that infection did impact overall survival (HR 2.28, 95% CI 1.14–4.55; *p*=0.019) and graft survival (HR 1.98, 95% CI 1.10–3.56; *p*=0.023). Figures 2(a) and 2(b) depict overall and graft survival by infection status.

**Figure 3:**
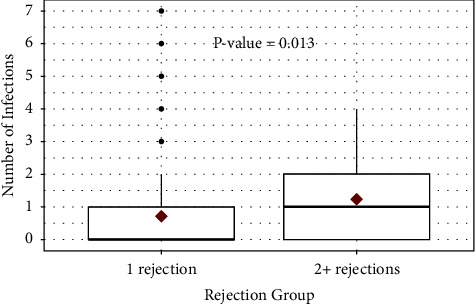
Number of infections by multiple rejections. The group of patients with ≥2 episodes of rejection had a significantly higher number of infections (*p*=0.013).

Given that there were 23 patients with more than one episode of rejection and subsequent treatment, we investigated if the cumulative addition of multiple episodes of rejection was a risk factor for subsequent infection development. We used a Wilcoxon rank sum test to compare two groups (one episode of rejection vs. 2 or more episodes of rejection) and found that patients with 2 or more episodes of rejection had significantly more infections than patients with just one episode of rejection (*p*=0.013) (Figure 3).

Finally, we investigated the impact of OIs on overall patient survival and allograft survival, using OI as a time-dependent covariate. We found a nonsignificant trend towards decreased patient survival (HR = 1.81, 95% CI 0.98–3.34 *p* = 0.059) but no effect on allograft survival (HR 1.51, 95% CI 0.85–2.68, *p* = 0.20).

## 4. Discussion

Infections are known to be a major cause of morbidity and mortality following kidney transplantation, with previous studies showing an association between post-transplant infections and allograft dysfunction [16, 24, 25]. However, there is limited research about infections that occur after treatment for KT rejection. Our group previously investigated this topic; we evaluated patients who did and did not develop infections after treatment for rejection and found that female gender, higher neutrophil count at the time of rejection, and increased number of rejection episodes were predictors of infection development after treatment for rejection [14]. We also found, similar to this study, that infections were predictors of graft loss in KT patients.

This current study aimed to understand the relationship between rejection and infections better. Since randomizing KT recipients to rejection and no rejection in a controlled trial is not plausible, we used propensity score matching, with multivariable statistical methods such as logistic regression, to evaluate the probability of developing an infection after rejection treatment and to reduce bias in estimating rejection effects and reduce the likelihood of confounding [26]. Previous studies have established that the analysis of a propensity score-matched sample can approximate that of a randomized control trial, thus is the best test for evaluating our topic of interest [27]. Here, we found that rejection treatment was a risk factor for infection development, with the rejection cohort having a higher cumulative incidence of infection than the nonrejection cohort. This has previously been assumed in clinical practice, and our study helps define this relationship with the proof of concept. While the absolute number of infections was higher in the control cohort than the case cohort due to a longer period of infection detection (18 months vs. 6 months), cumulative incidence of infection was higher in the case cohort. The case cohort had a faster time to infection after development of rejection than the controls, with a comparable total duration of follow-up between both groups (508.9 days for cases vs 526.8 days for controls). We hypothesize that the higher cumulative incidence of infection in the cases is due to the augmented immunosuppression patients receive after an episode of rejection. The association between rejection and infection was further confirmed with multivariate analysis.

We did analyze in a prior study whether any specific rejection therapy in KT patients is an independent predictor of infection in our cohort. We found that no specific rejection therapy, including ATG, IV steroids, IVIG, or plasmapheresis, was on its own a predictor of infection, thus eliminating this as a confounder [14].

Similar to our previous study as well, an increased number of rejection episodes and their associated treatments were a risk factor for a higher number of infections. Additionally, we found that the presence of OIs resulted in a trend toward a higher rate of patient mortality. The lack of statistical significance in this population was likely due to the small number of total OIs in the study, resulting in low power of this subgroup. When using time-dependent Cox regression to analyze our data, the above conclusions were found to be the same, further strengthening our results.

The most frequent infections noted in this study were UTIs, which is in concordance with data reported from prior studies [17, 28]. Viral pathogens, particularly CMV, accounted for the second highest proportion of infections. Even though there was a higher percentage of CMV mismatch in the controls compared to the cases, there was no difference in the incidence of CMV infections (disease and viremia) postrejection between the groups. This suggests that rejection and its associated treatment is likely a risk factor for CMV infection, and prophylaxis for this OI is of utmost importance.

Surprisingly, our data showed that rejection did not predict allograft survival or overall survival, which contrasts with previous research [29, 30]. We acknowledge that this conclusion has not been previously supported in the literature; however, we suspect that our study's finding is multifactorial. At our institution, after an episode of acute transplant rejection is treated, immunosuppression is optimized and oral steroids are added to the maintenance regimen if patients were not already on it. Additionally, some patients receive enhanced immunosuppression with eculizumab, rituximab, antithymocyte globulin, and others, in an effort to treat and prevent further episodes of rejection. The strict postrejection protocols in place at our institution may explain this outcome. Additionally, since we matched eGFR at four months to better assess the impact of infection, patients with severe rejection and poor allograft function following rejection are possibly not included in this study (the average matched eGFR at four months in this study was 48). Thus, CKD 4 and 5 patients' postrejection were not included in our study. Furthermore, we evaluated graft survival at 6 months, and the rate of graft loss may have been higher if we expanded the time frame to 12 or 24 months.

Although our study did not reveal an association between rejection and survival, it did show that infections following treatment for KT rejection adversely affected allograft survival and overall patient survival, in agreement with a previous study that identified a 1.91-fold higher risk for graft failure and 2.22-fold higher mortality risk associated with infections in KT patients [17]. This has important implications for clinical practice. Clinicians should promptly identify and treat these infections, especially UTIs and OIs, and patients who develop infections after rejection treatment may need to be monitored more closely and more frequently. Additionally, these results stress the importance of resuming OI-specific prophylaxis, especially if an episode of rejection occurs after 6 months of posttransplant.

We recognize limitations of this study. The duration of follow-up for infection detection in cases was limited; however, infections related to rejection treatment should be identified in a 6-month time frame. We also had a small number of true OIs, likely due to our strict infection prophylaxis protocols. Additionally, the diverse nature of infections evaluated in this study introduces variability in the clinical impact. However, we calculated that 41.0% of infections in the case cohort required hospitalization, while 27.2% of the infections in the control group required hospitalization, underscoring the clinical impact of infections in KT patients who have experienced rejection. Third, patients in this study were matched on their eGFR at 4 months of posttransplant, and 68 patients in the rejection cohort had an episode of rejection prior to four months. However, when these patients were excluded from the analysis, the results of our study did not change. Additionally, those episodes of rejection were likely not severe enough to cause kidney injury or an increase in baseline creatinine as compared to the controls. As mentioned previously, there were a higher total number of infections in the control cohort, likely due to longer duration of potential infection detection, but cumulative incidence of infection was significantly higher in the case cohort. Fifth, as we can see from Table 2, the majority (80.5%) of patients in the study had TCMR with lower numbers of the other types of infection; thus, it is hard to evaluate the impact of AMR and mixed types independently. However, our objective was to evaluate the impact of rejection as a whole. Furthermore, while we did match for select comorbidities, we did not address all etiologies for renal failure, and we acknowledge that these can impact baseline health status and susceptibility to rejection and infection. Lastly, we acknowledge the limitations associated with retrospective studies as well as the inability of propensity score matching to completely eliminate selection bias or account for unobserved variables.

In summary, we found that kidney transplant rejection is a predictor of subsequent infection development, and those infections negatively impact allograft survival and overall patient survival. These infections in KT patients treated for rejection also frequently result in hospitalization, emphasizing their clinical impact. Clinicians should have a low threshold to thoroughly investigate for infections in KT patients who are treated for rejection, as they can have long-term deleterious effects. Additionally, these data emphasize the need for close and heightened surveillance of KT patients in the postrejection period. Furthermore, based on our data, we believe that recycling of prophylaxis after rejection treatment is of high importance in preventing postrejection infections and decreasing overall mortality and graft loss. A larger, prospective study is needed to better understand this relationship and develop guidelines for treatment of such patients.

## Figures and Tables

**Table 1:** Demographics and clinical characteristics for control and case cohorts.

	Case (*n* = 185)	Control (*n* = 185)	*p* value
Age at transplant			0.657^1^
Mean (SD)	53.6 (14.0)	53.0 (14.6)	
Male			0.675^2^
No	82 (44.3%)	78 (42.2%)	
Yes	103 (55.7%)	107 (57.8%)	
Diabetes			0.596^2^
No	108 (58.4%)	113 (61.1%)	
Yes	77 (41.6%)	72 (38.9%)	
BMI			0.428^1^
Mean (SD)	28.6 (5.9)	28.1 (5.5)	
Txp year			0.878^2^
2014	31 (16.8%)	33 (17.8%)	
2015	24 (13.0%)	26 (14.1%)	
2016	23 (12.4%)	18 (9.7%)	
2017	26 (14.1%)	21 (11.4%)	
2018	35 (18.9%)	33 (17.8%)	
2019	35 (18.9%)	44 (23.8%)	
2020	11 (5.9%)	10 (5.4%)	
Deceased donor			0.202^2^
No	34 (18.4%)	44 (23.8%)	
Yes	151 (81.6%)	141 (76.2%)	
Induction therapy			0.948^2^
Basiliximab	67 (36.2%)	69 (37.3%)	
Alemtuzumab	104 (56.2%)	101 (54.6%)	
Antithymocyte globulin	14 (7.6%)	15 (8.1%)	
Steroid avoidance			0.746^2^
No	119 (64.3%)	116 (62.7%)	
Yes	66 (35.7%)	69 (37.3%)	
eGFR at 4 months			0.935^1^
Mean (SD)	49.7 (20.1)	49.5 (19.3)	

The baseline demographics and clinical characteristics for both cohorts, after matching. There were no significant differences between the cohorts. Analysis completed using ^1^two-sample *t*-test and ^2^Pearson's chi-squared test.

**Table 2:** Infections.

Infection type	Case (*n* = 169)	Control (*n* = 297)	Total (*n* = 466)
Abscess	7 (4.1%)	1 (0.3%)	8 (1.7%)
Bacteremia	14 (8.3%)	23 (7.7%)	37 (7.9%)
BK nephropathy	4 (2.4%)	3 (1.0%)	7 (1.5%)
BK viremia	9 (5.3%)	37 (12.5%)	46 (9.9%)
Candida	3 (1.8%)	2 (0.7%)	5 (1.1%)
Clostridioides difficile associated disease	9 (5.3%)	10 (3.4%)	19 (4.1%)
CMV disease	3 (1.8%)	0 (0.0%)	3 (0.6%)
CMV viremia	24 (14.2%)	40 (13.5%)	64 (13.7%)
EBV viremia	2 (1.2%)	6 (2.0%)	8 (1.7%)
Herpes simplex (disseminated)	1 (0.6%)	0 (0.0%)	1 (0.2%)
JC polyoma virus	0 (0.0%)	1 (0.3%)	1 (0.2%)
VZV (localized)	2 (1.2%)	0 (0.0%)	2 (0.4%)
Mold infection	1 (0.6%)	0 (0.0%)	1 (0.2%)
Mycobacteria	0 (0.0%)	1 (0.3%)	1 (0.2%)
Nocardia	1 (0.6%)	4 (1.3%)	5 (1.1%)
Other	11 (6.5%)	24 (8.1%)	35 (7.5%)
Pneumocystis jirovecii pneumonia (PJP)	0 (0.0%)	1 (0.3%)	1 (0.2%)
Pneumonia requiring admission	6 (3.6%)	13 (4.4%)	19 (4.1%)
Pulmonary coccidioidomycosis	2 (1.2%)	1 (0.3%)	3 (0.6%)
Pyelonephritis	10 (5.9%)	9 (3.0%)	19 (4.1%)
Symptomatic UTI	60 (35.5%)	121 (40.8%)	181 (38.9%)

All infections identified in this study, with a breakdown between the case and control cohorts. Other infections: parvovirus (4), norovirus (4), coronavirus (not SARS-CoV-2) upper respiratory tract infection (4), rhinovirus (3), parainfluenza virus (2), salmonella gastroenteritis (2), bacillus cereus colitis (2), unspecified colitis (2), influenza virus URI (1), west nile virus meningitis (1), human papilloma virus (1), hepatitis B (1), unspecified osteomyelitis (1), necrotizing fasciitis (1), human metapneumovirus (1), RSV bronchitis (1), orchitis (1), cellulitis (1), legionella pneumonia (1), enteropathogenic E. coli gastroenteritis (1).

## Data Availability

The data that support the findings of this study are available upon request from the corresponding author. The data are not publicly available due to privacy or ethical restrictions.

## Abbreviations

TCMR: T-cell mediated rejection
HLA: Human leukocyte antigens
AMR: Antibody-mediated rejection
DSA: Donor-specific antibodies
g: Glomerulitis
ptc: Peritubular capillaritis
OPTN: Organ procurement and transplant network
EHR: Electronic health record
PHS: Public health service
EBV: Epstein–Barr virus
CMV: Cytomegalovirus
eGFR: Estimated glomerular filtration rate
OI: Opportunistic infections
REDCap: Research electronic data capture
BMI: Body mass index
IVIG: Intravenous immunoglobulin
HR: Hazards ratio
CI: Confidence interval
VZV: Varicella zoster virus
IV: Intravenous
KT: Kidney transplant
PLEX: Plasmapheresis
ATG: Antithymocyte globulin.

## Supplementary Materials

Supplementary Materials Supplemental Figure 1: standardized mean difference. We have calculated the standardized difference for each of the variables (mean for continuous variables and prevalence for categorical variables) that were used to calculate the propensity score to assess the balance of the variables between the two groups before and after propensity score matching. From the figure, we can see that propensity score matching reduced the imbalance between the two groups to a large degree, especially for transplant year, eGFR at 4 months, and steroid avoidance.
