# Supplementary material for: Impact of Early Rejection Treatment on Infection Development in Kidney Transplant Recipients: A Propensity Analysis
Source: J Transplant. 2024 Mar 1;2024:6663086. doi: 10.1155/2024/6663086 (PMC10923621; doi:10.1155/2024/6663086)
Supplement: Supplementary Materials — Supplemental Figure 1: standardized mean difference. We have calculated the standardized difference for each of the variables (mean for continuous variables and prevalence for categorical variables) that were used to calculate the propensity score to assess the balance of the variables between the two groups before and after propensity score matching. From the figure, we can see that propensity score matching reduced the imbalance between the two groups to a large degree, especially for transplant year, eGFR at 4 months, and steroid avoidance. [file 6663086.f1.docx]

Supplemental Figure 1


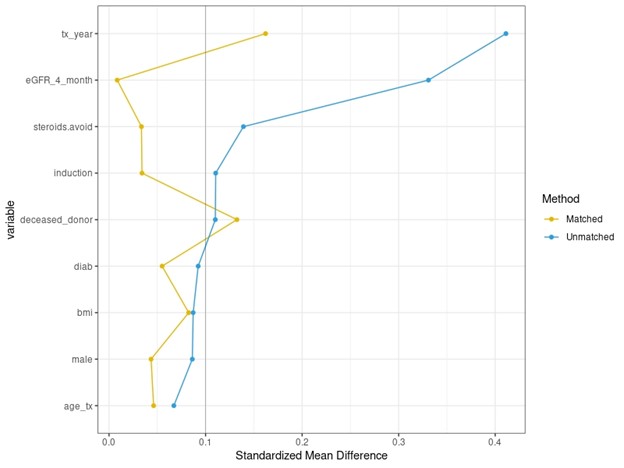


Supplemental Figure 1. Standardized mean difference. We have calculated the standardized difference for each of the variables (mean for continuous variables and prevalence for categorical variables) that were used to calculate the propensity score to assess the balance of the variables between the two groups before and after propensity score matching. From the figure, we can see that propensity score matching reduced the imbalance between the two groups to a large degree especial for transplant year, eGFR at 4 month and steroid avoidance.
